# Supplementary material for: The inositol 5-phosphatase INPP5B regulates B cell receptor clustering and signaling
Source: J Cell Biol. 2022 Jul 25;221(9):e202112018. doi: 10.1083/jcb.202112018 (PMC9351708; doi:10.1083/jcb.202112018)
Supplement: Table S1 — lists the source and catalogue numbers for the reagents and antibodies used in this study. [file JCB_202112018_TableS1.docx]

**Table S1**

| **Reagent** | **Supplier** | **Identifier** |
| --- | --- | --- |
| RPMI-1640 | Sigma-Aldrich | R8758 |
| RPMI-1640 (no phenol red) | Thermo Scientific | 10363083 |
| Heat-inactivated fetal bovine serum | Thermo Scientific | 16140071 |
| Chicken Serum | Sigma-Aldrich | C5405 |
| Penicillin-Streptomycin | Sigma-Aldrich | P4333 |
| Blasticidin S HCl | Thermo Scientific | R21001 |
| Puromycin dihydrochloride | abcam | ab141453 |
| G418 Sulfate | Thermo Scientific | 10131035 |
| Hygromycin B (50mg/ml) | Thermo Scientific | 10453982 |
| 2-Mercaptoethanol (50 mM) | Thermo Scientific | 11528926 |
| Protease Inhibitor Cocktail | Sigma-Aldrich | P8340 |
| Protein assay dye reagent | BioRad | 5000006 |
| Anti-FLAG M2 affinity gel | Sigma-Aldrich | A2220 |
| Anti-6X His tag® antibody (Rabbit) | abcam | Ab9108 |
| Indole-3-acetic acid sodium salt (auxin) | Sigma-Aldrich | **I5148** |
| Goat polyclonal anti-chicken IgM | BioRad | AA127 |
| Goat anti Human IgM mu chain | Abcam | ab97201 |
| Mouse anti-chicken IgM M1 clone | SouthernBiotech | 8310-01 |
| Mouse monoclonal M-4 Anti-Chicken IgM mu chain | abcam | ab99715 |
| LysoTracker™ Green DND-26 | ThermoScientific | L7526 |
| Donkey anti-Mouse IgG antibody, HRP | Sigma-Aldrich | AP192P |
| Pierce® Goat anti-Rabbit IgG (H+L), HRP | Thermo Scientific | 31460 |
| Rabbit anti-Goat IgG (H+L), HRP | Thermo Scientific | 31402 |
| Texas Red®-X protein labeling kit | Thermo Scientific | T-10244 |
| Pierce™ Fab fragmentation kit | Thermo Scientific | 44985 |
| Raf1-RBD agarose beads | Sigma-Aldrich | 14-278 |
| Anti-Ras antibody (Clone RAS10) (mouse) | EMD Millipore | 05-516 |
| PAK1-PBD (agarose free) | Sigma-Aldrich | 14-864 |
| CDC42 antibody (Rabbit) | Cell Signaling Technology | 2462 |
| RalGDS-RBD^GST^ | Jena Bioscience | PR-365 |
| Rap1 (E-6) (mouse) | Santa Cruz Biotechnology | sc-398755 |
| Electroporation cuvettes | BioRad | 1652088 |
| Phosphatase Inhibitor Cocktail (100X) | Cell Signaling Technology | 5870 |
| Latrunculin A | abcam | ab144290 |
| ML 141, CDC42 GTPase inhibitor | Abcam | ab145603 |
| CK 666, actin polymerization inhibitor | Abcam | ab141231 |
| YU142670 compound  (3-(Pyridin-4-yl)-[1,2,4]triazolo[3,4-b][1,3,4]thiadiazole, 3-(4-pyridinyl)-1,2,4-Triazolo[3,4-b][1,3,4]thiadiazole) | Custom-synthesized | https://chem-space.com/ |
| Anti-GFP antibody (Rabbit) | abcam | ab290 |
| GAPDH antibody (Mouse) | Santa Cruz Biotechnology | sc-365062 |
| Actin antibody (Rabbit) | Cytoskeleton, Inc. | AAN01 |
| Alexa Fluor™ 488 Phalloidin | Thermo Scientific | A12379 |
| Ezrin antibody (Rabbit) | abbexa | Abx008576 |
| Phospho-Ezrin (Thr567) (Rabbit) | antibodies-online.com | ABIN2994843 |
| Rabbit polyclonal to Cofilin 2 (phospho S3) | abcam | \| ab14134 \|  \| \| --- \| --- \| |
| Phospho-Akt (Thr308) (D25E6) XP® Rabbit mAb | Cell Signaling Technology | 13038 |
| Phospho-Akt (Ser473) (D9E) XP® Rabbit mAb | Cell Signaling Technology | 4060 |
| Akt Antibody | Cell Signaling Technology | 9272 |
| p44/42 MAPK (Erk1/2) (137F5) Rabbit mAb | Cell Signaling Technology | 4695 |
| Phospho-Syk (Tyr525/526) antibody (Rabbit) | Cell Signaling Technology | 2711 |
| Syk (D3Z1E) XP® Rabbit mAb | Cell Signaling Technology | 13198 |
| Phospho-FoxO1 (Ser256) antibody (Rabbit) | Cell Signaling Technology | 9461 |
| FoxO1 (L27) antibody (Rabbit) | Cell Signaling Technology | 9454 |
| Human Phospho-NFATc1 (S172) antibody (mouse) | R&D systems | MAB5640 |
| Human NFATc1 antibody (Goat) | R&D systems | AF5640 |
| Anti-OCRL (Rabbit) | Sigma-Aldrich | HPA012495 |
| µ-Dish 35 mm, high Glass Bottom | Ibidi | 81158 |
| µ-Slide 4 Well Glass Bottom | Ibidi | 80427 |
| Poly-L-lysine solution | Sigma-Aldrich | P8920 |
| Zeba Spin 7K MWCO Desalting Columns | Thermo Scientific | 10415545 |
| UltraPure™ BSA (50mg/ml) | Thermo Scientific | AM2616 |
| Dynasore analog | abcam | ab120192 |
| Pierce PES Protein Concentrators | Thermo Scientific | 11355402 |
| SuperSignal™ West Femto Maximum Sensitivity Substrate | Thermo Scientific | 34095 |
| m-3M3FBS | Tocris Bioscience | \| 1941 \|  \| \| --- \| --- \| |
| Protein A Agarose (High Affinity) | abcam | \| ab193255 \|  \| \| --- \| --- \| |
| HEPES, 1M Buffer Solution | Thermo Scientific | 11550496 |
| FITC Annexin V Apoptosis Detection Kit | BioLegend® | 640914 |
| EasySep™ Human B Cell Isolation Kit | STEMCELL™ Technologies | 17954 |
