# Supplementary material for: The inositol 5-phosphatase INPP5B regulates B cell receptor clustering and signaling
Source: J Cell Biol. 2022 Jul 25;221(9):e202112018. doi: 10.1083/jcb.202112018 (PMC9351708; doi:10.1083/jcb.202112018)
Supplement: Table S2 — lists the sequences for the primers used in this study. [file JCB_202112018_TableS2.docx]

**Table S2**

| **Oligo name** | **Sequence (5`-3`)** |
| --- | --- |
| 5`ARM-INPP5B-F | AGATATCGATCATTCGGAATCTTGTGAGTTA |
| 5`ARM-INPP5B-R | ACATGAATTCCGATAAATTGTTCTCTCAG |
| PCR-Degron-F | ATATGAATTCGACGGAGCTGGT |
| PCR-Degron-R | ATATGGATCCCGGGCCGCTAGTGAT |
| 3`ARM-INPP5B-F | GCTAGGTACCATTTCAGATGCAGCTAATCT |
| 3`ARM-INPP5B-R | GACGTCTAGAGTGGTTTTCTCAAGAACATGGT |
| SDM-INPP5B-F | GGCCTGTAGGGATCATGAGGGTCAC |
| SDM-INPP5B-R | CTCCAGTGACCCTCATGATCCCTAC |
| PCR-m3`-INPP5B-F | ATAGGATCCATTTCAGATGCAGCTAAT |
| PCR-m3`-INPP5B-R | ATATGCGGCCGCGTGGTTTTCTCAAGAACA |
| SCR-pINPP5B-AID-F | CTGAGATCACAATTGAGTTGGAAC |
| SCR-AMD1-R | CATGAGTCGTAGATCCTTCCTTG |
| Test-INPP5B-F | ATCTGATGGCATTCCTACGAGAA |
| Test-INPP5B-R | CAGCTTATTAACAGTGACACCTG |
| PCR-5`ARM-OCRL-F | ATATGTCGACAGAGATCAGCTGGGCTTAAA |
| PCR-5`ARM-OCRL-R | ATATGAATTCCTCCTCATCCCCGGCAAGCA |
| PCR-3`ARM-OCRL-F | ATATGTCGACCTCCTGCCTCTTGCCAGACTG |
| PCR-3`ARM-OCRL-R | ATGAGAATTCCTCTGCAGGAAGCACTGATGT |
| SDM-OCRL-F | CGGATCTGGGTGGATTCGCTGATAGC |
| SDM-OCRL-R | GCTCAGCTATCAGCGAATCCACCCAG |
| PCR-m3`-OCRL-F | ATATGGATCCCTCCTGCCTCTTGCCAG |
| PCR-m3`-OCRL-R | ATATGCGGCCGCAGCACAGCACTATAGGGCAG |
| SCR-AMD1-F | ACATGTCCTTGACTGTACGTC |
| 5`-INPP5B-KI-F-01 | ATATGGTACCGCTTCATATGTGACACTGATGT |
| 5`-INPP5B-KI-R-01 | AGATATCGATCTGCAAATTGTTCACAGAG |
| 5`-INPP5B-KI-F-02 | ATATGTCGACGCTTCATATGTGACACTGATGT |
| 5`-INPP5B-KI-R-02 | ATATGGATCCCTGCAAATTGTTCACAGAG |
| 3`-INPP5B-KI-F | ATATGGATCCTTGAACAAGAAGCTGGCTAGC |
| 3`-INPP5B-KI-R | AGTCTCTAGATCACGATAAATTGTTCTCT |
| SDM-INPP5B-1-F | TTGCTGGATCATGAGGAGATCTGAGA |
| SDM-INPP5B-1-R | GATCTCCTCATGATCCAGCAATAAAG |
| SDM-INPP5B-2-F | GGCTCTGAAGATCAGTGGACACAAGCCAGTTA |
| SDM-INPP5B-2-R | AGAACTAACTGGCTTGTGTCCACTGATCTTCA |
| SCR-SDM-Integ-F | GCATACATTATACGAACGGTAGG |
| SCR-SDM-Integ-R | GCTGCATCTGAAATTCACGAT |
| SCR-SDM-KI-F | ACACAAGGTACAGTGGTTGCTTC |
| SCR-SDM-KI-R | ATGTATGCTATACGAACGGTAGG |
| PCR-EGFP-Tubby-F | ATATGGATCCATGGTGAGCAAGGGCGAGGA |
| PCR-EGFP-Tubby-R | ATATGCGGCCGCTTACTCGCAGGCCAGCTTG |
| PCR-Lifeact-EGFP-F | ATATGGATCCATGGGCGTGGCCGACCTGAT |
| PCR-Lifeact-EGFP-R | ATATGCGGCCGCTTACTTGTACAGCTCGTCCATGC |
