# Supplementary figures and images for: The inositol 5-phosphatase INPP5B regulates B cell receptor clustering and signaling
Source: J Cell Biol. 2022 Jul 25;221(9):e202112018. doi: 10.1083/jcb.202112018 (PMC9351708; doi:10.1083/jcb.202112018)

# Panel A

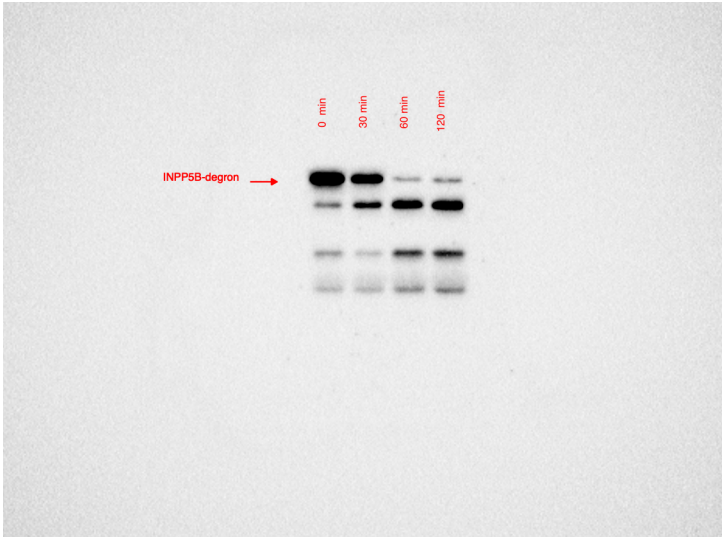

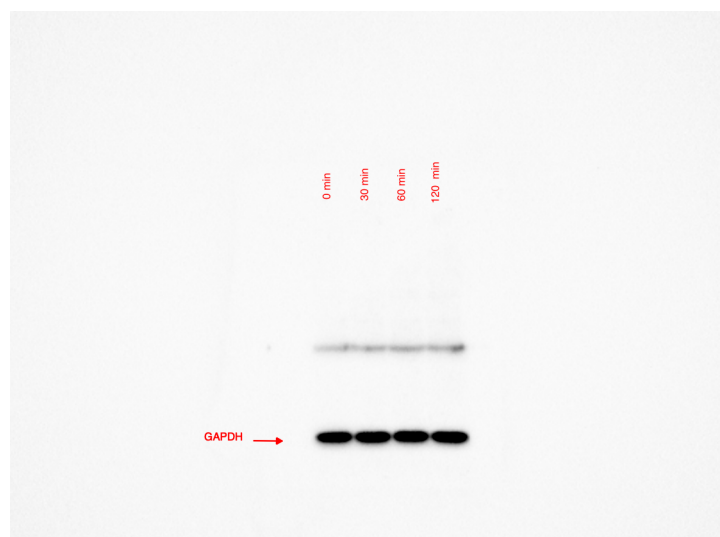

Supplement: SourceData F1 — is the source file for Fig. 1. [file JCB_202112018_SourceDataF1.pdf]

Panel A

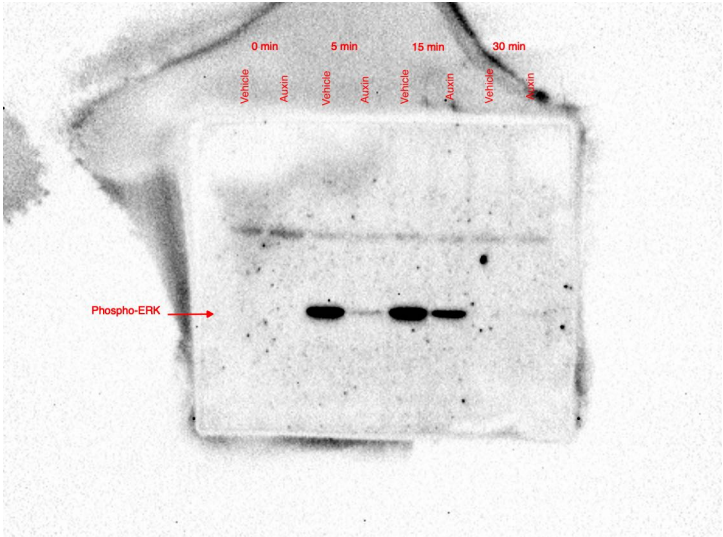

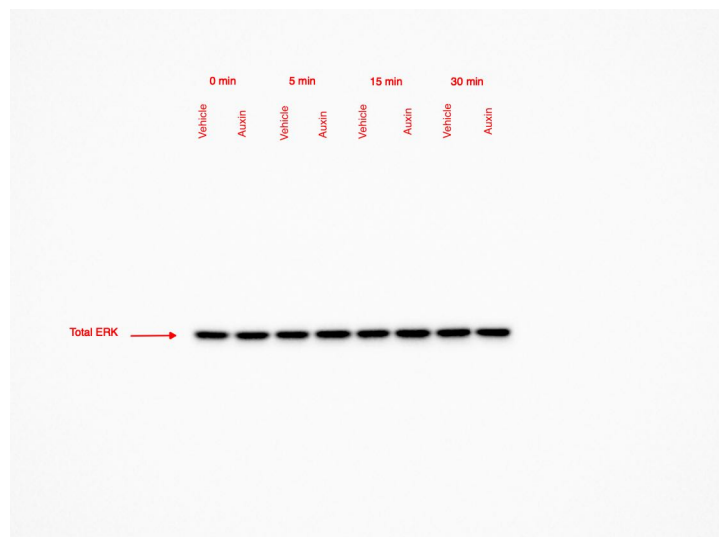

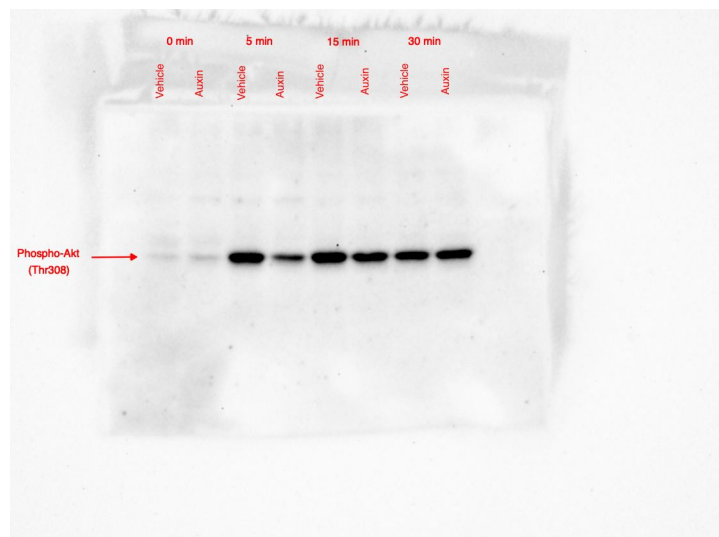

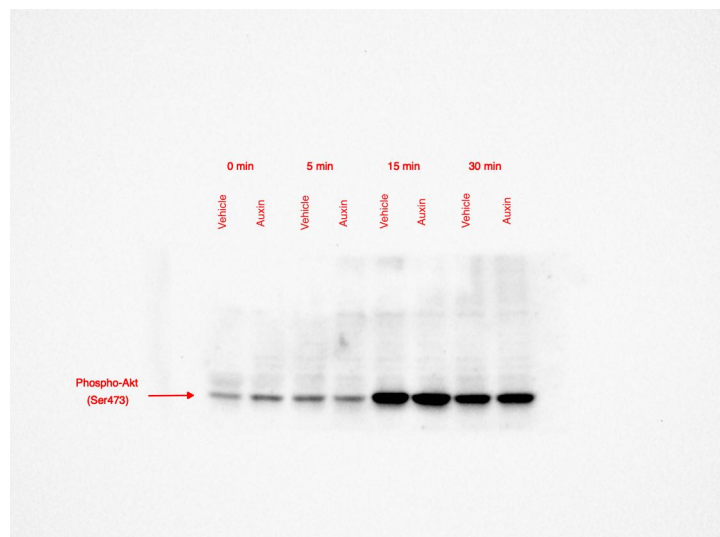

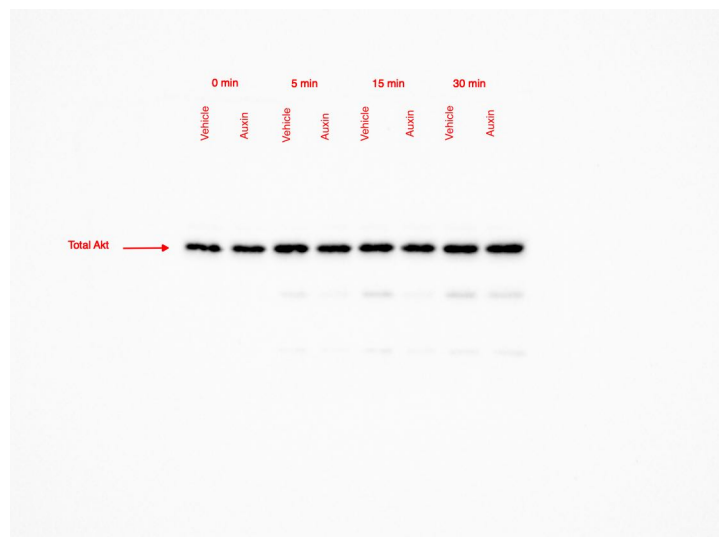

# Panel B

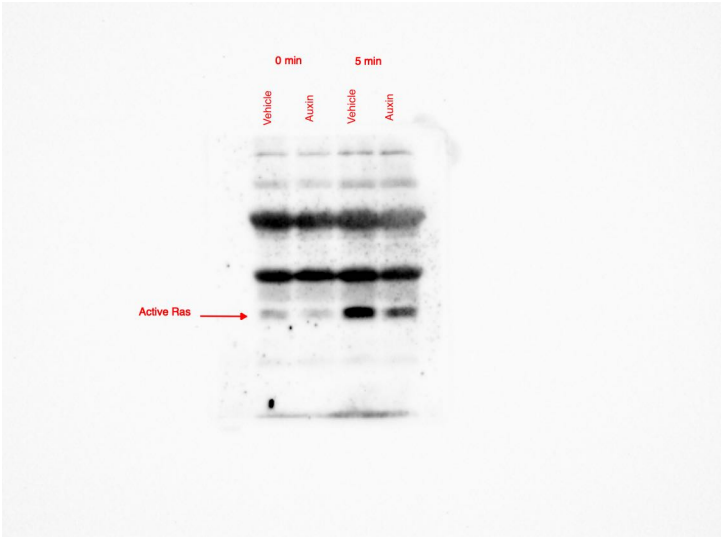

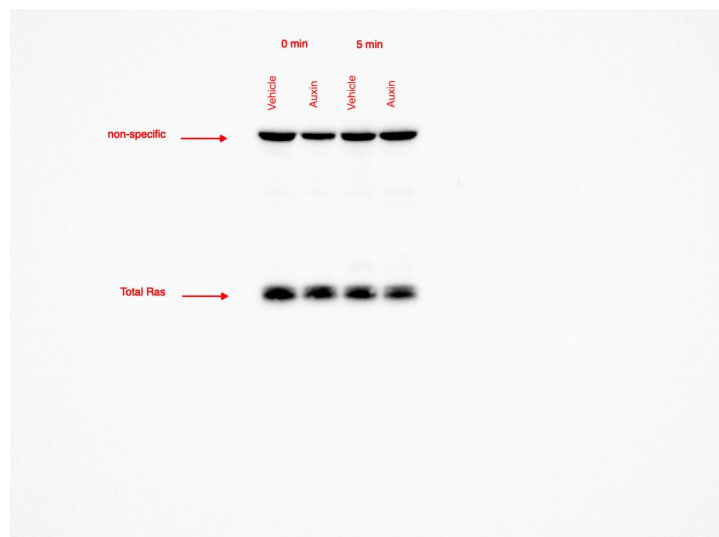

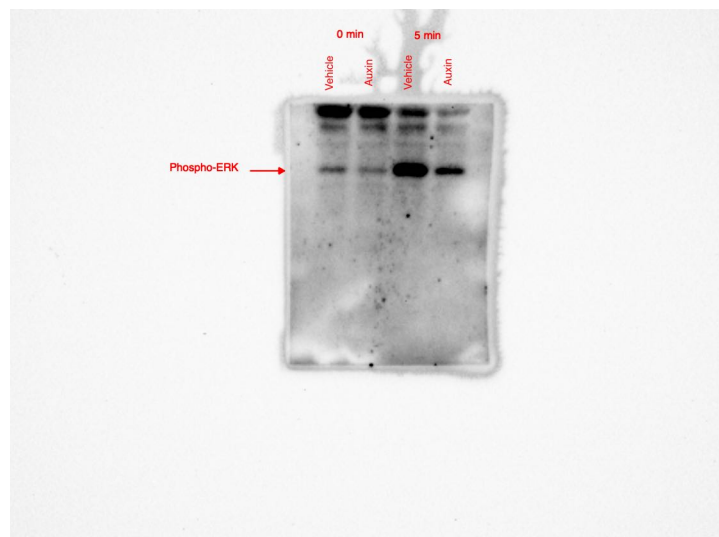

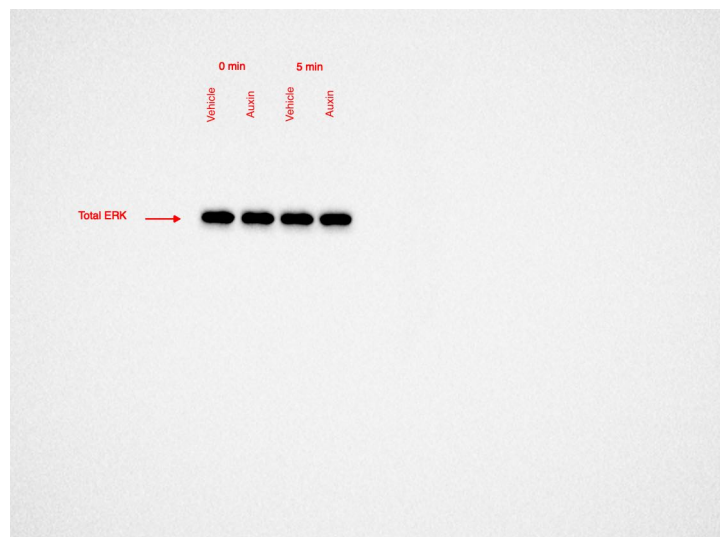

# Panel C

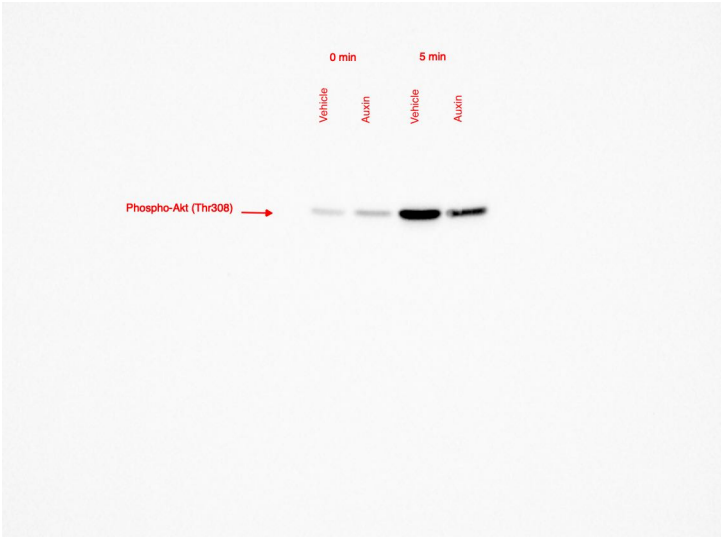

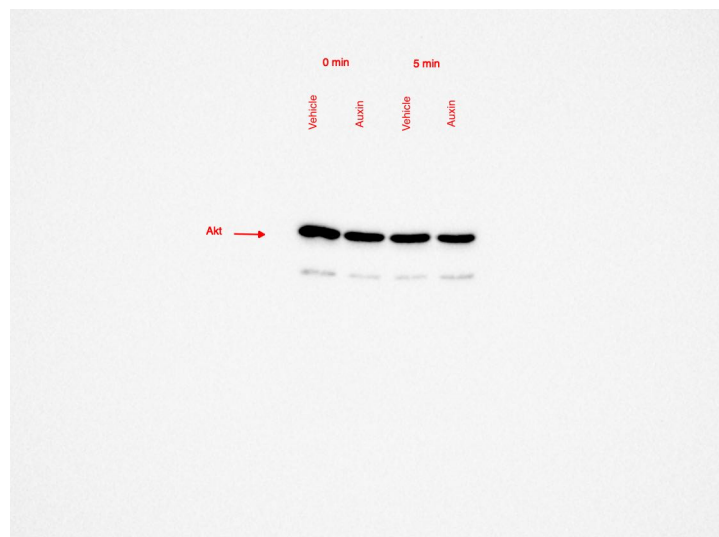

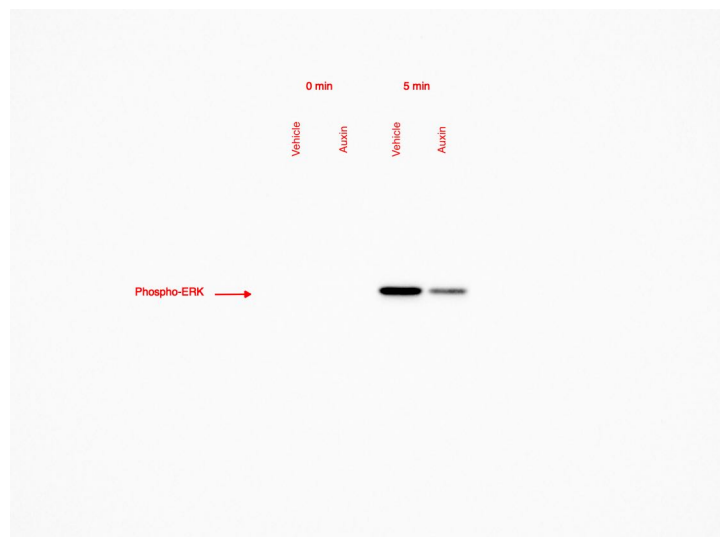

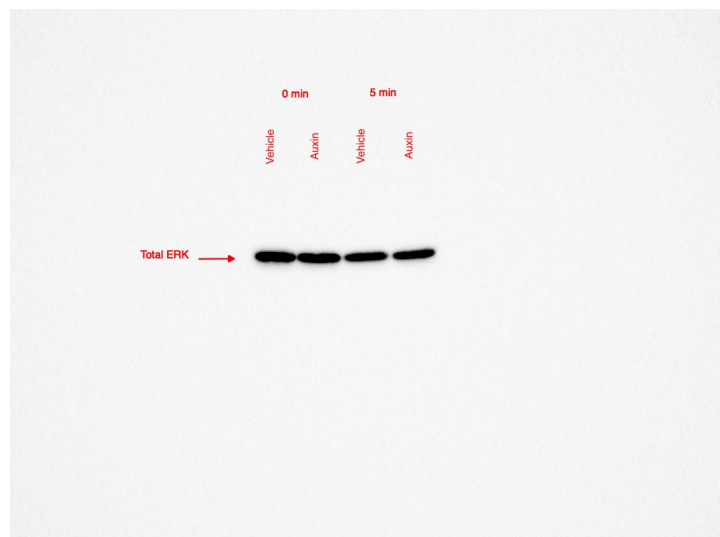

Panel E

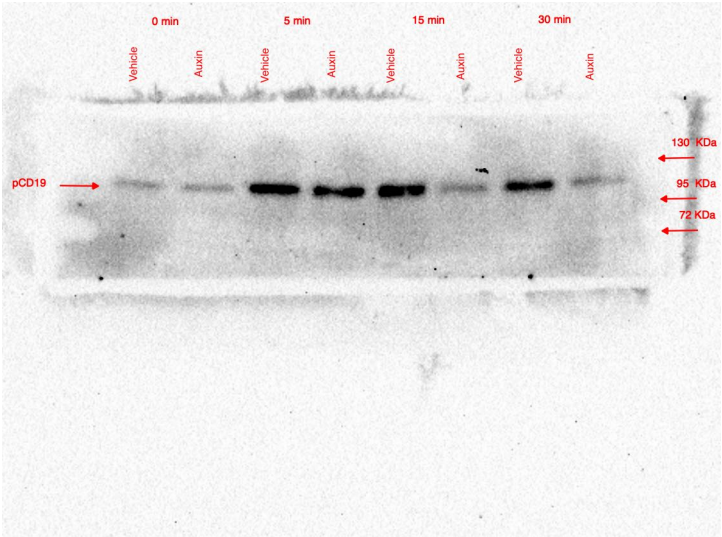

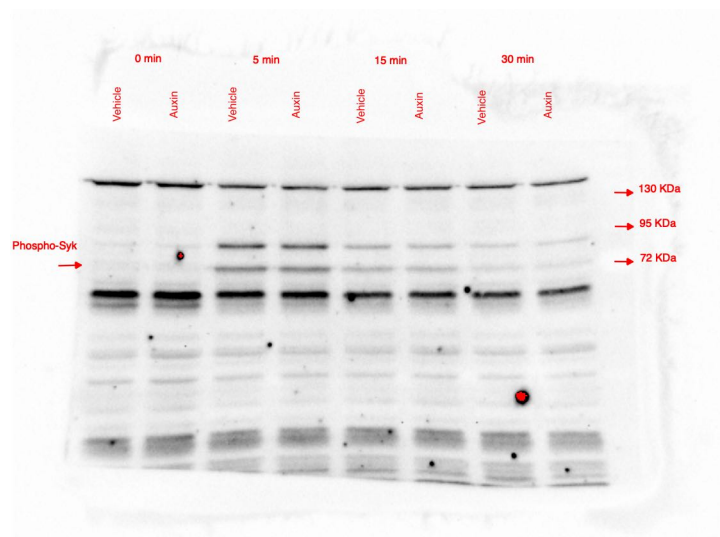

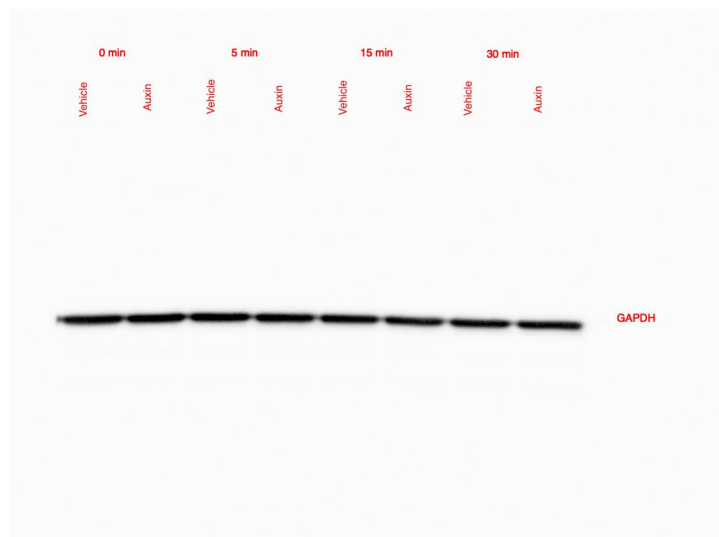

Supplement: SourceData F2 — is the source file for Fig. 2. [file JCB_202112018_SourceDataF2.pdf]

Panel A

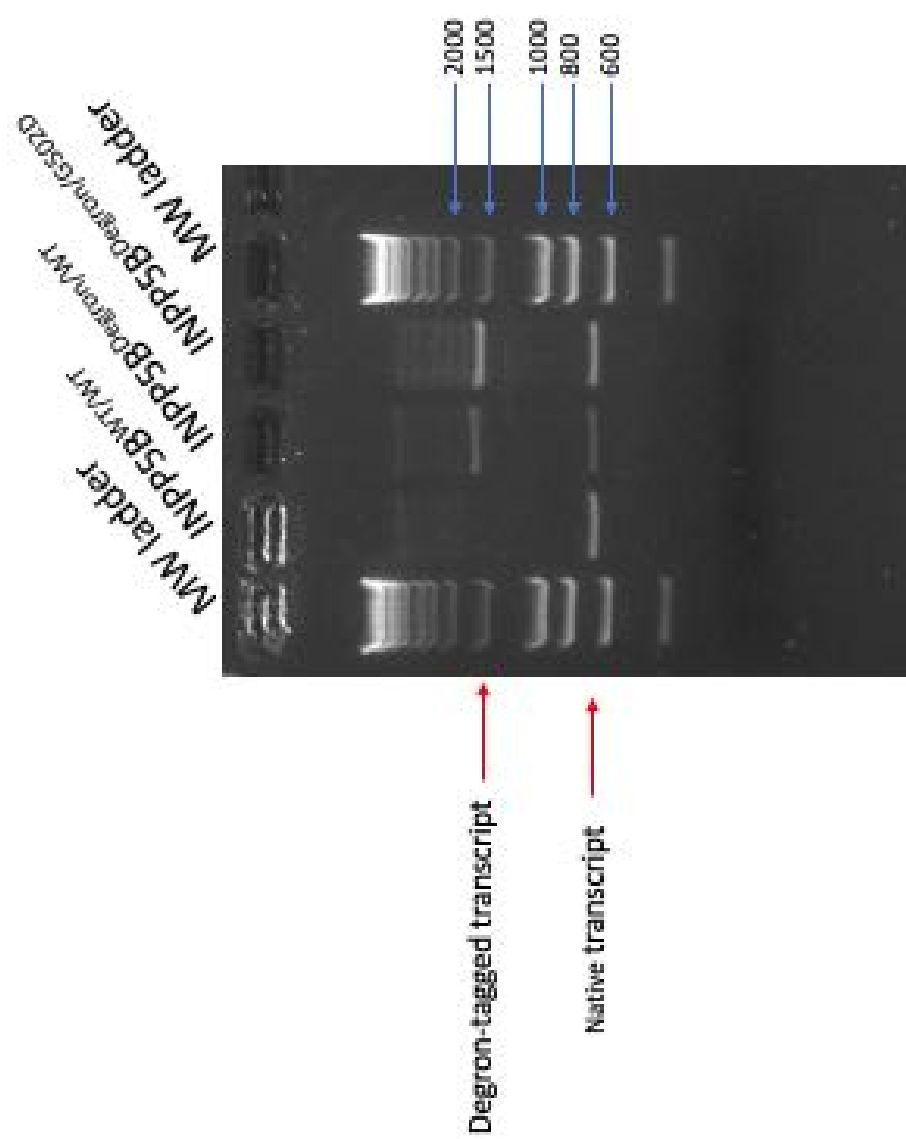

# Panel I

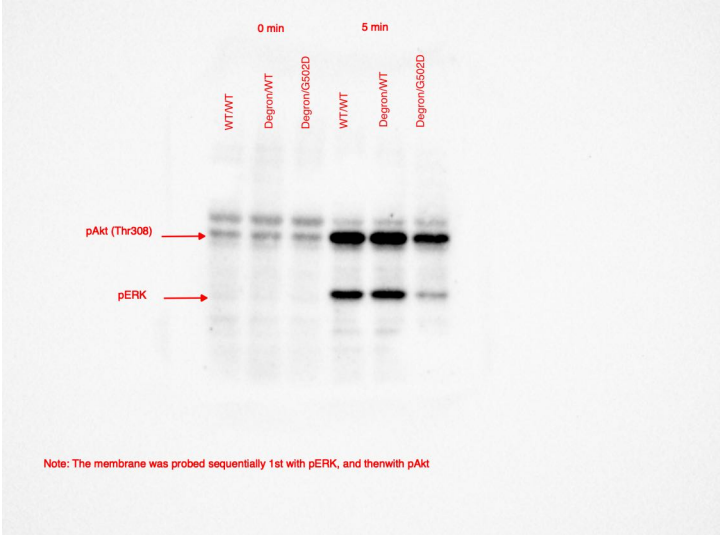

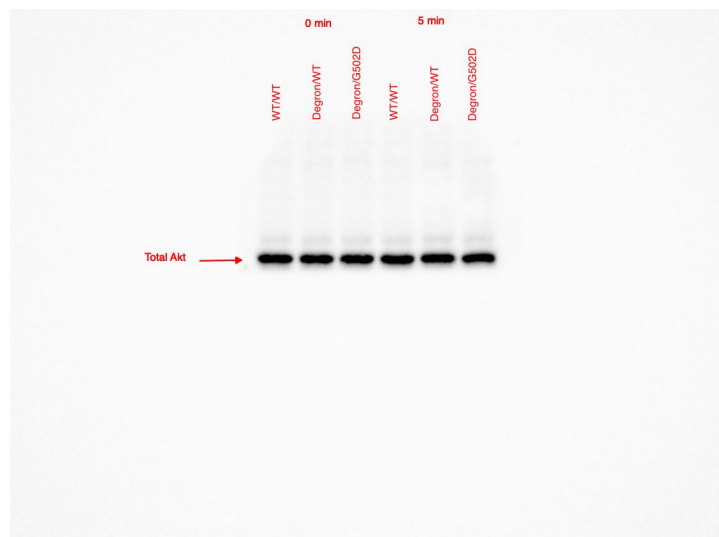

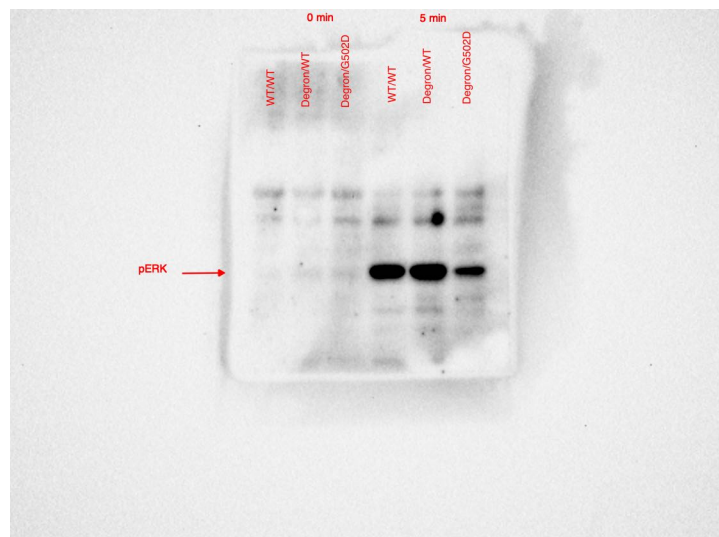

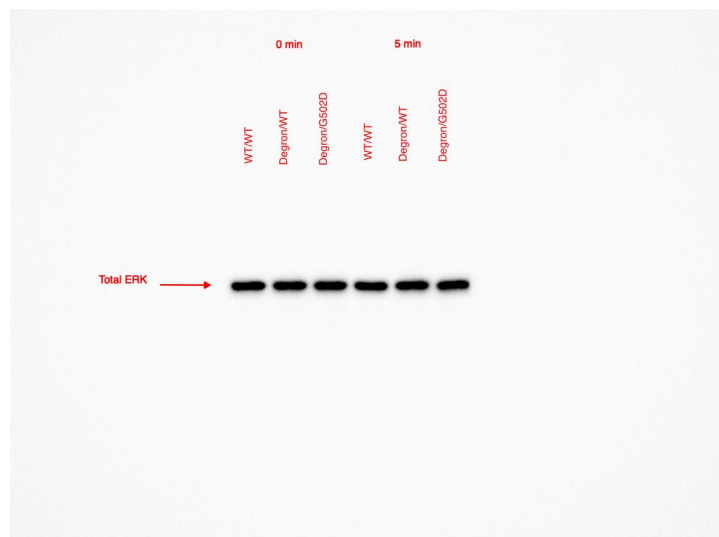

# Panel J

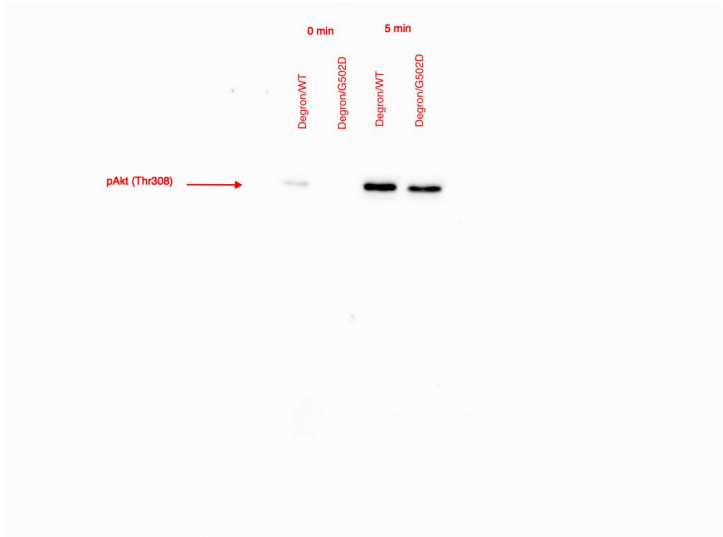

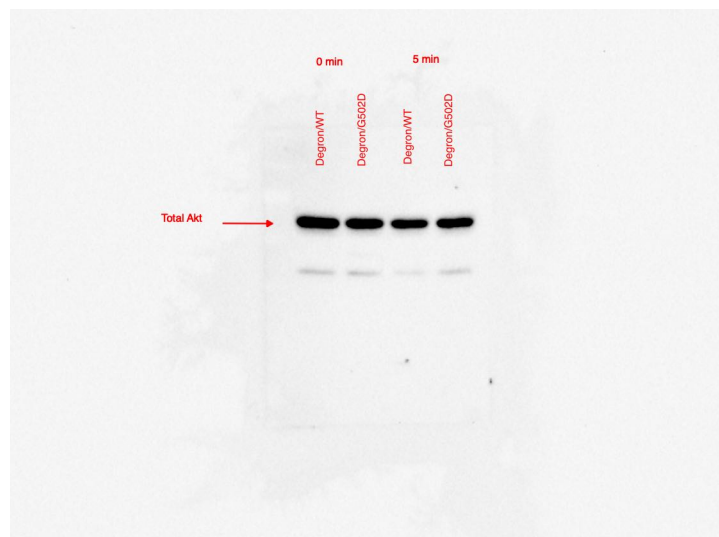

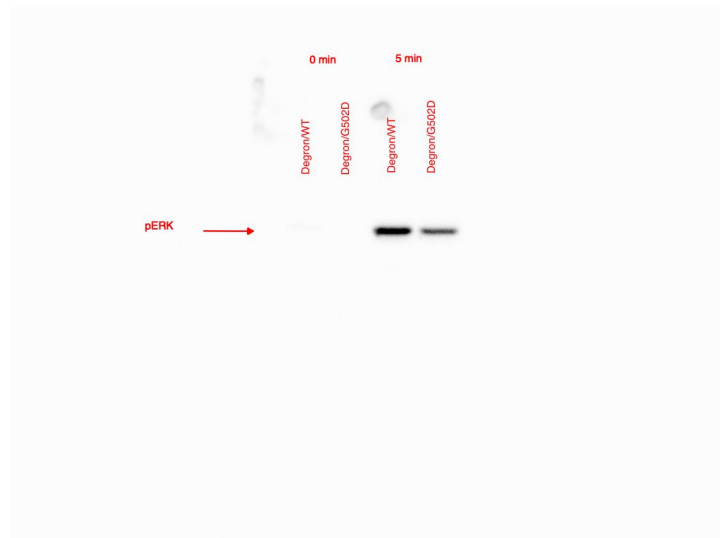

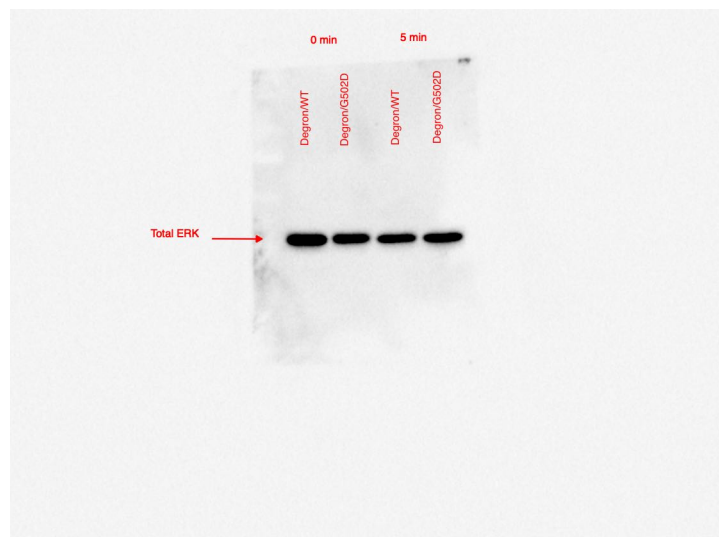

Supplement: SourceData F3 — is the source file for Fig. 3. [file JCB_202112018_SourceDataF3.pdf]

# Panel C

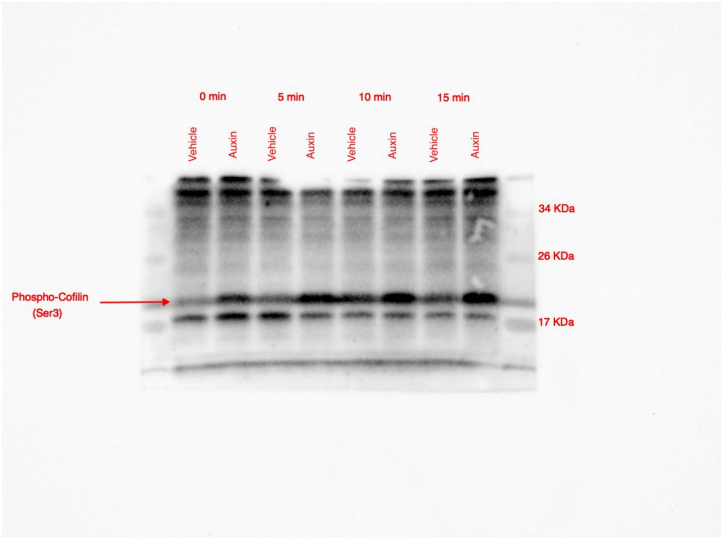

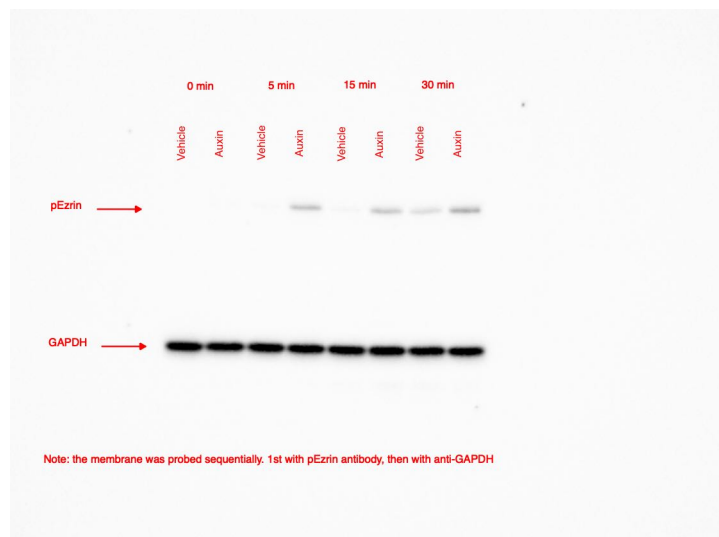

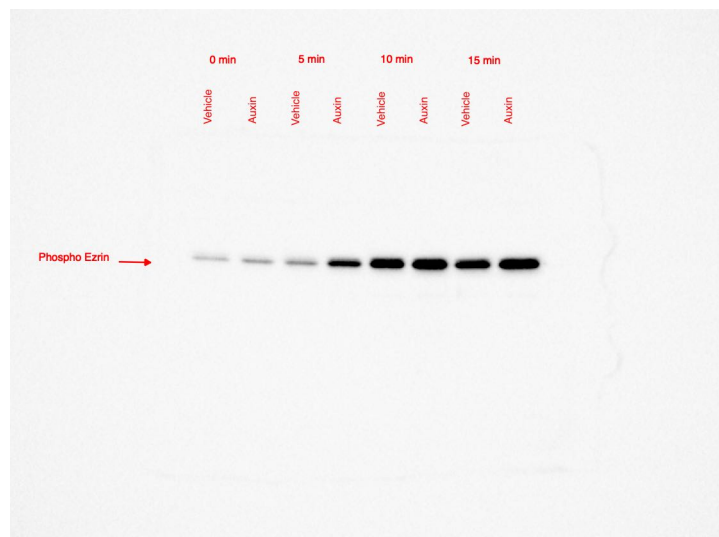

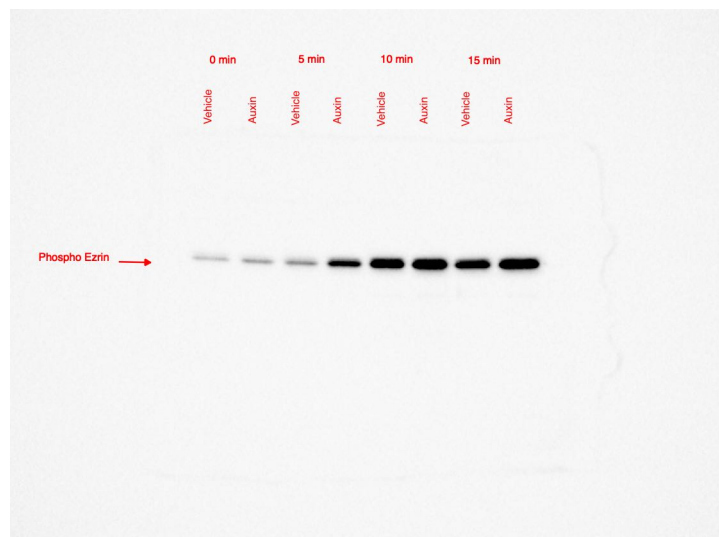

# Panel E

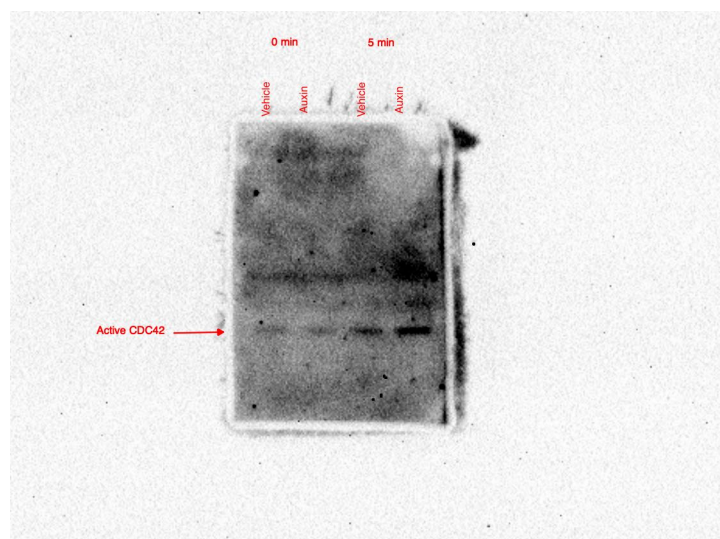

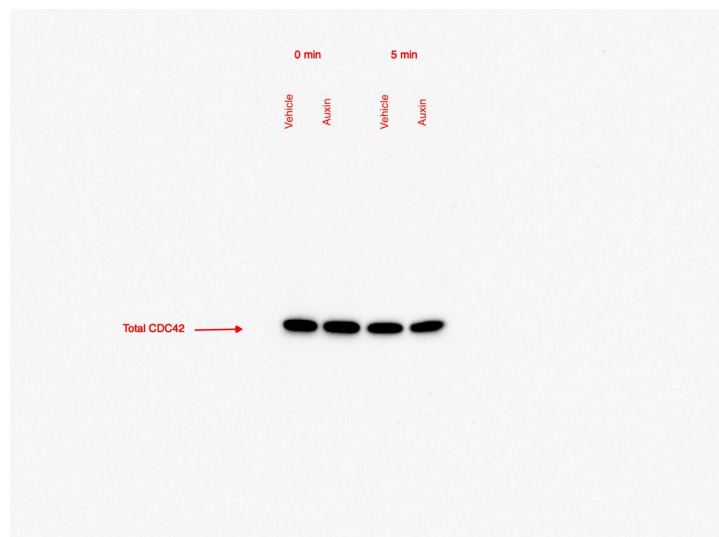

Supplement: SourceData F6 — is the source file for Fig. 6. [file JCB_202112018_SourceDataF6.pdf]

# Panel C

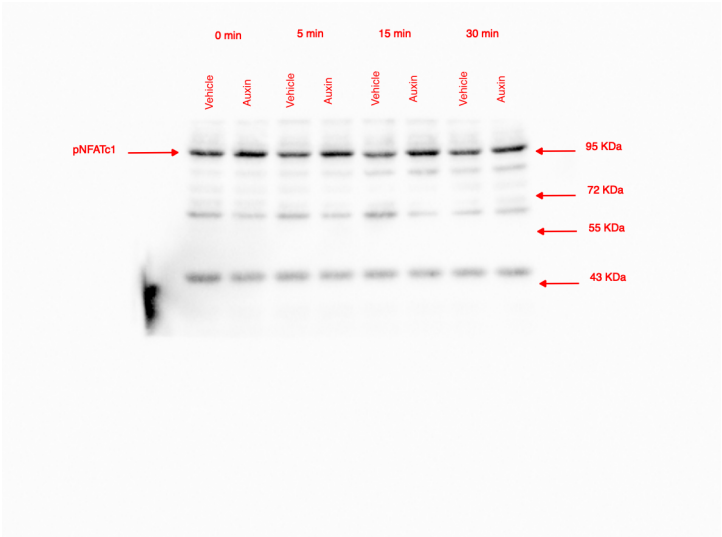

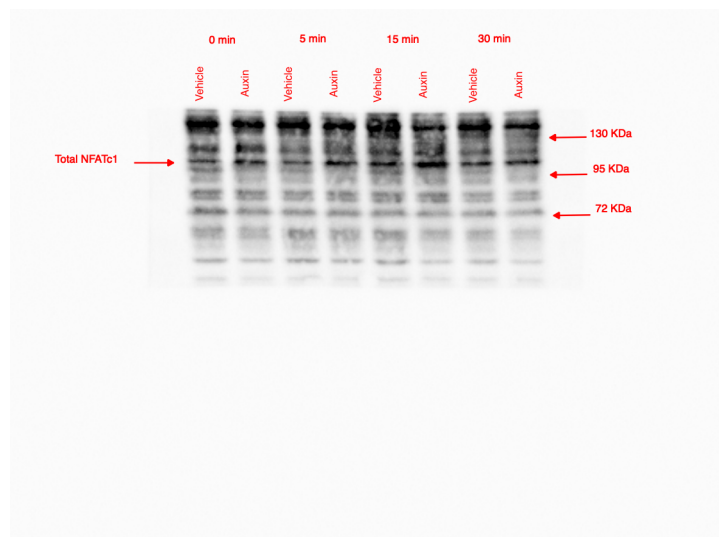

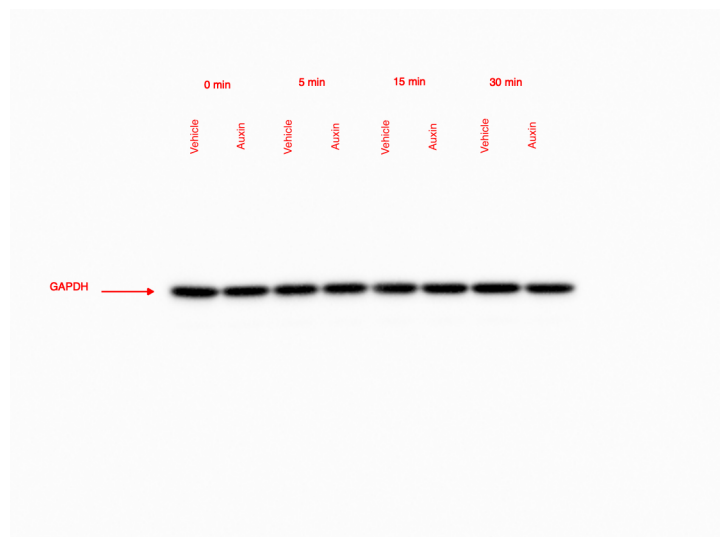

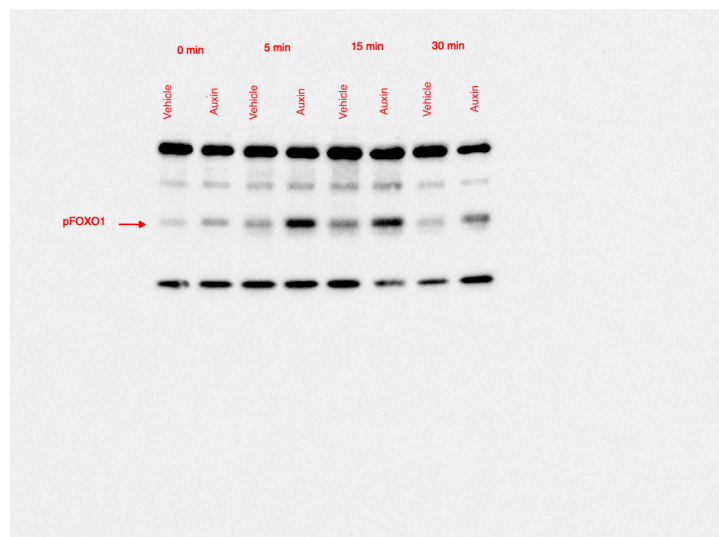

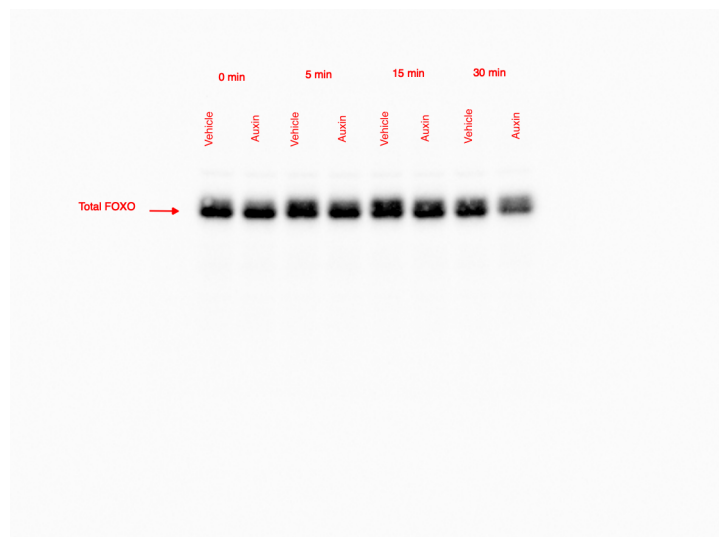

Supplement: SourceData F7 — is the source file for Fig. 7. [file JCB_202112018_SourceDataF7.pdf]

# Panel A

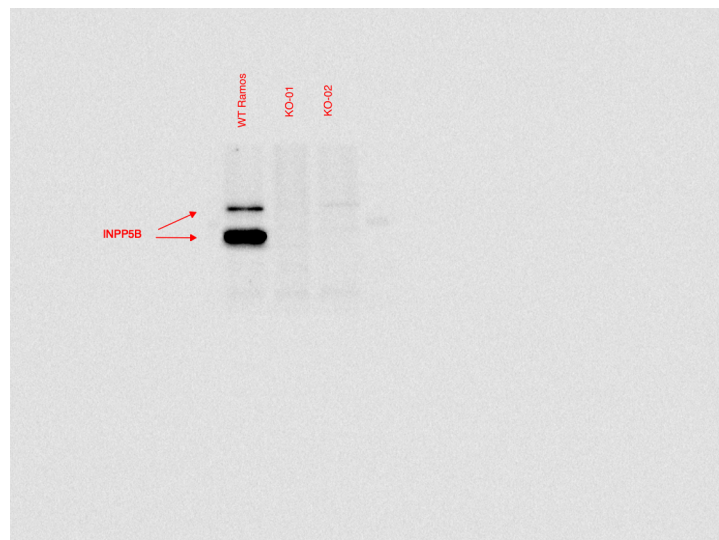

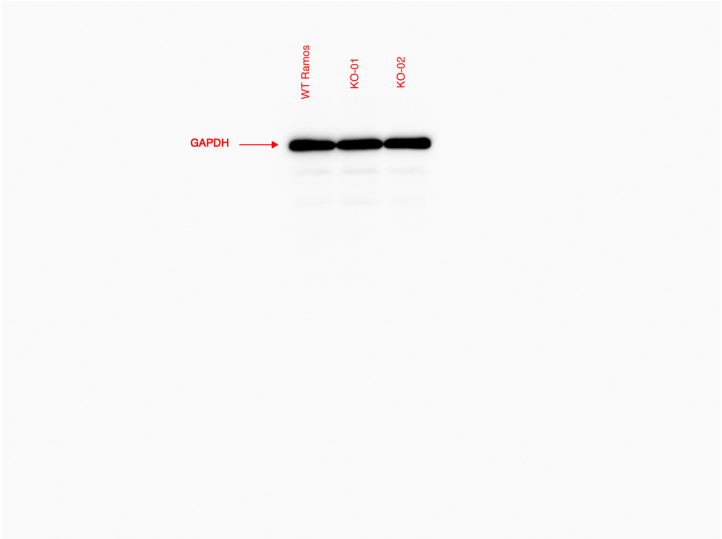

Supplement: SourceData F8 — is the source file for Fig. 8. [file JCB_202112018_SourceDataF8.pdf]

Panel B

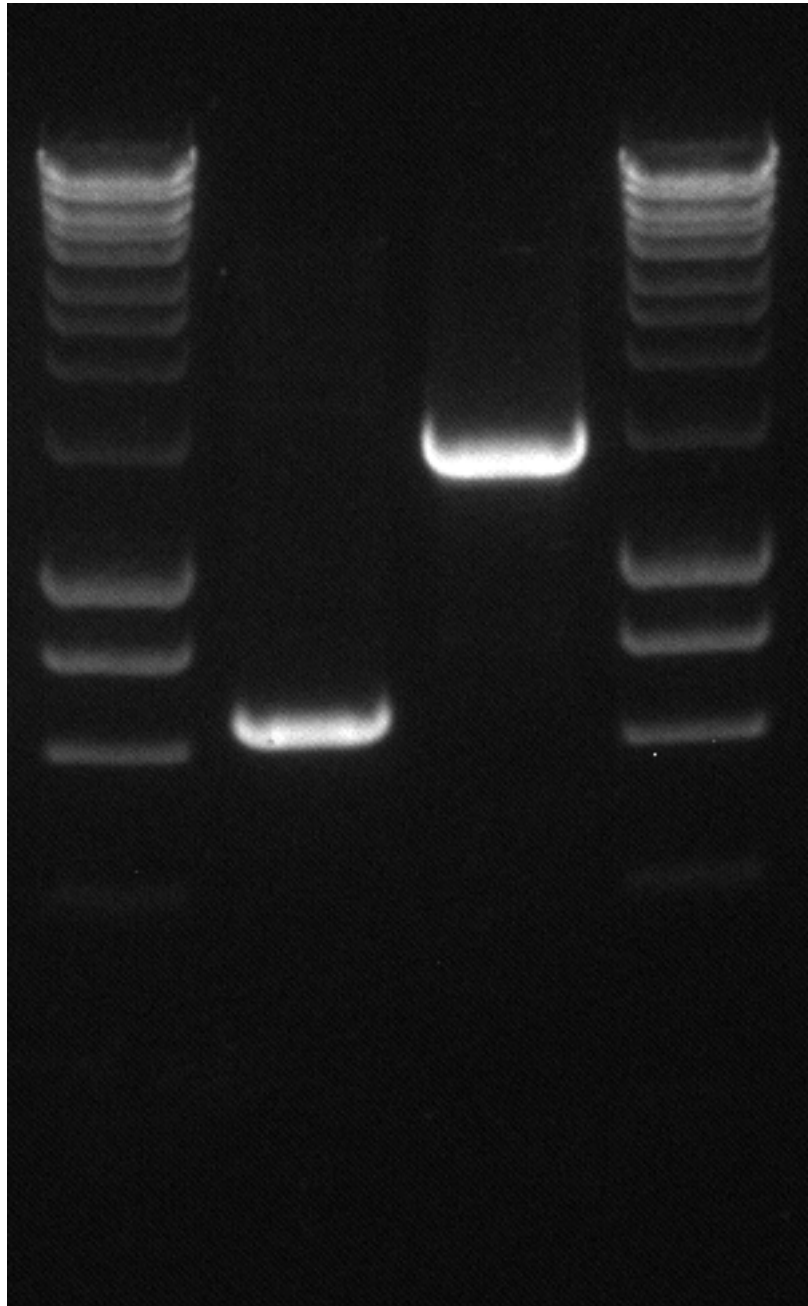

## Panel C

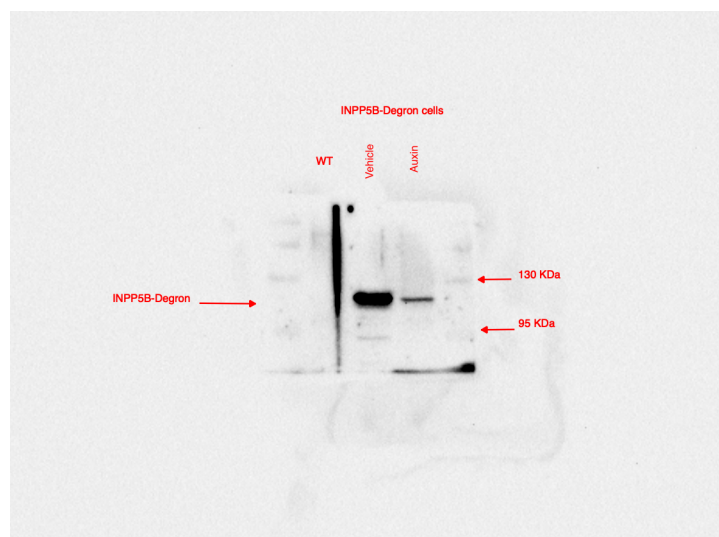

Supplement: SourceData FS1 — is the source file for Fig. S1. [file JCB_202112018_SourceDataFS1.pdf]

Panel A

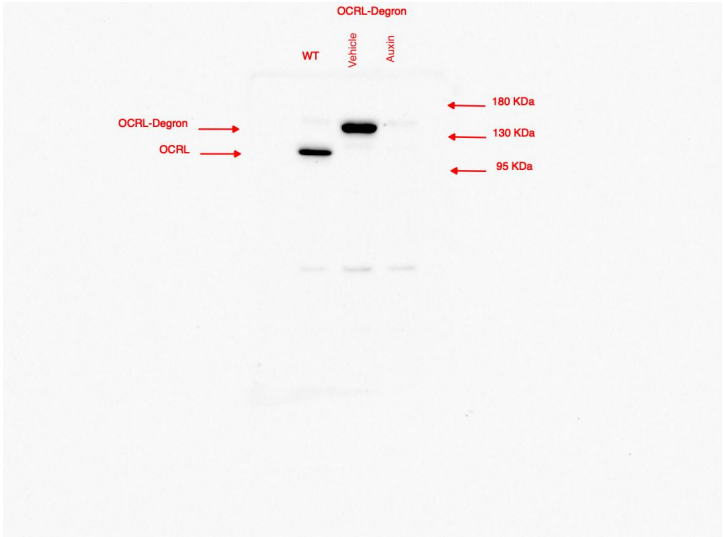

## Panel I

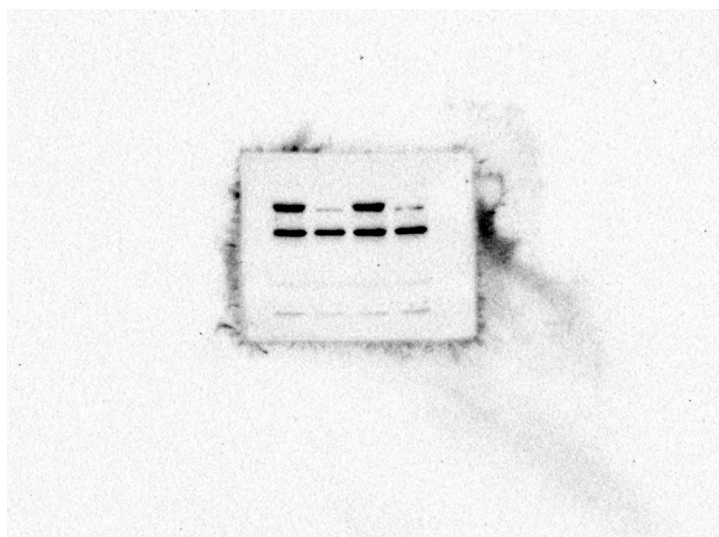

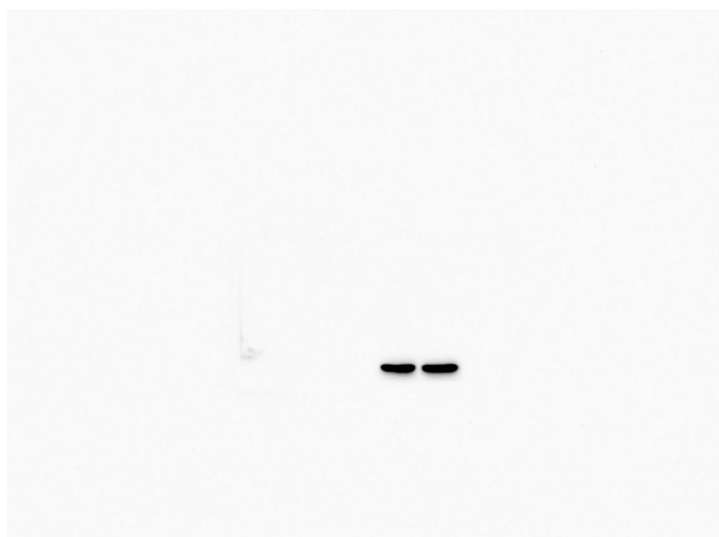

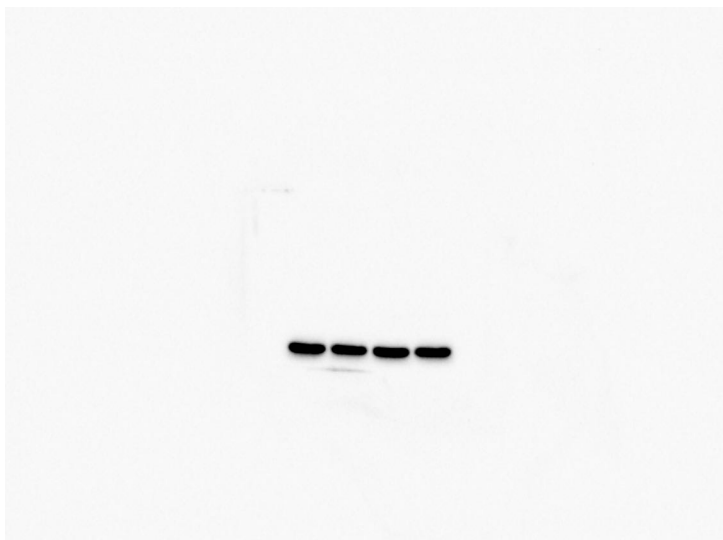

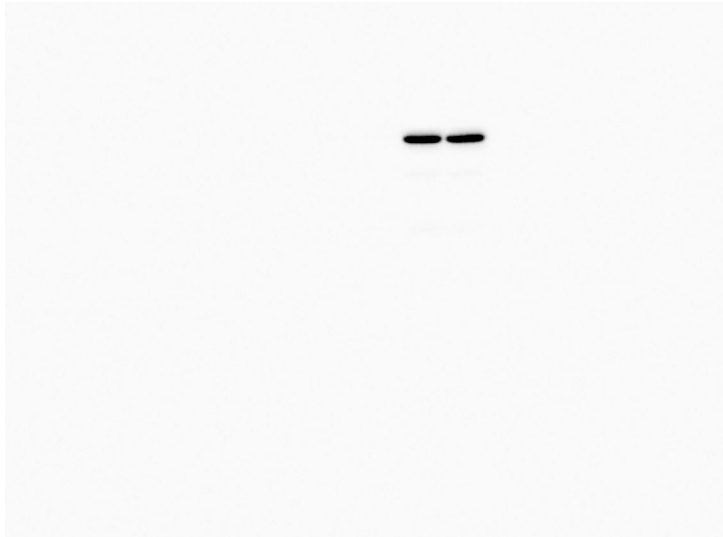

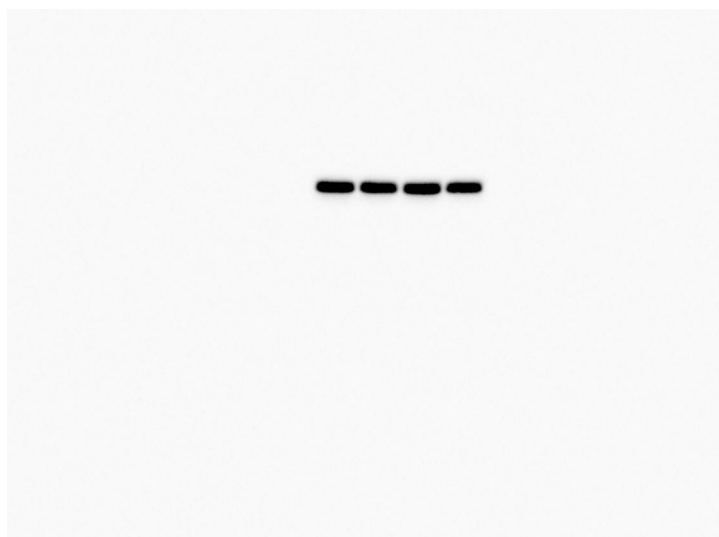

Supplement: SourceData FS2 — is the source file for Fig. S2. [file JCB_202112018_SourceDataFS2.pdf]

## Panel C

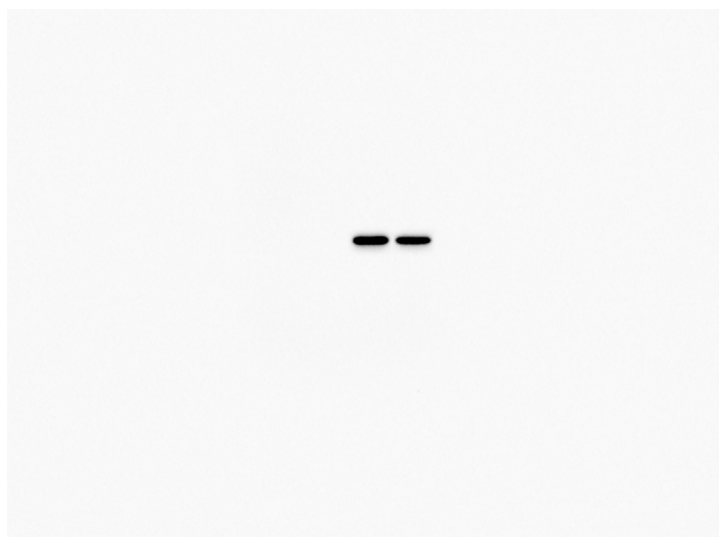

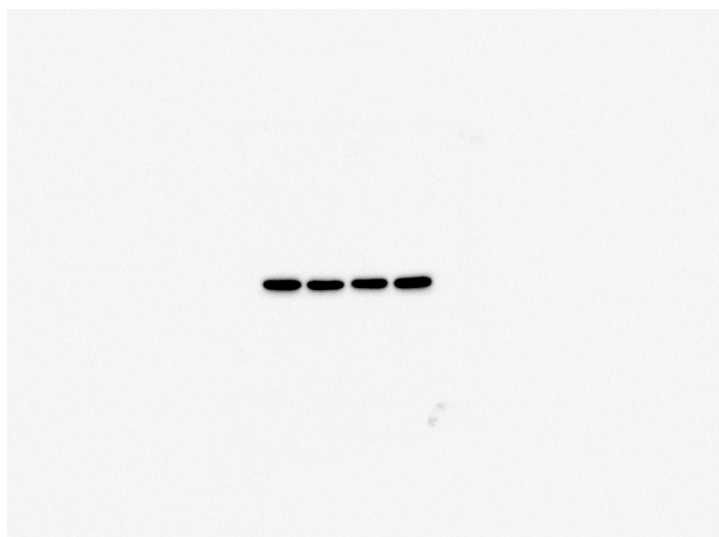

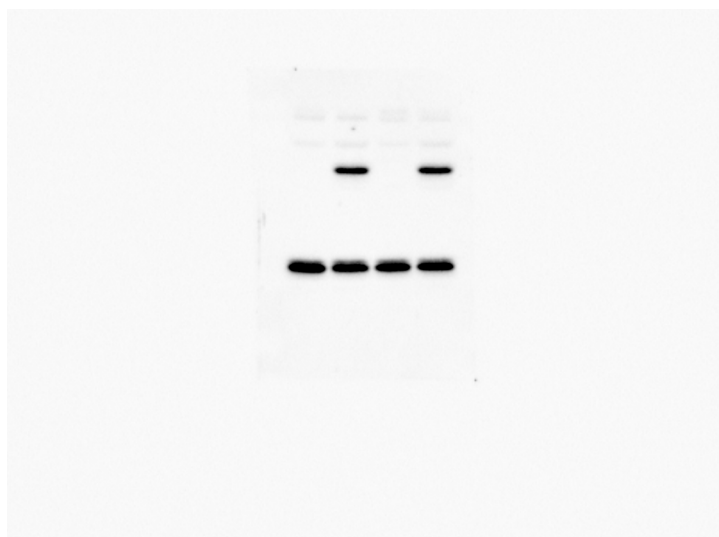

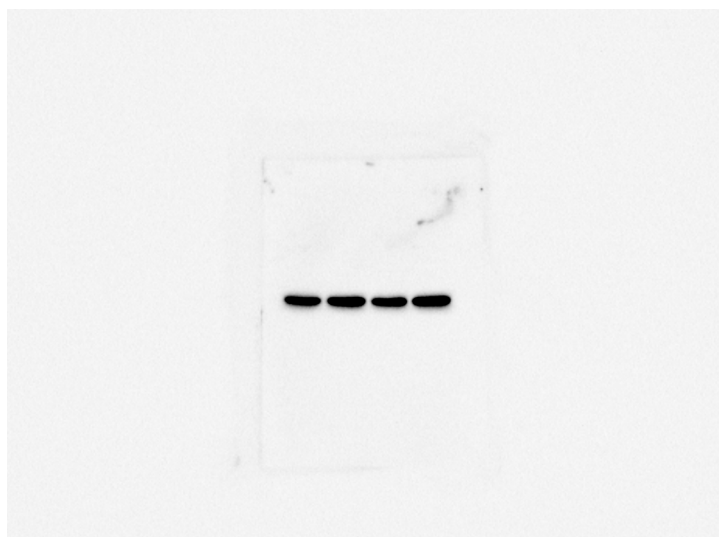

Supplement: SourceData FS3 — is the source file for Fig. S3. [file JCB_202112018_SourceDataFS3.pdf]

## Panel C

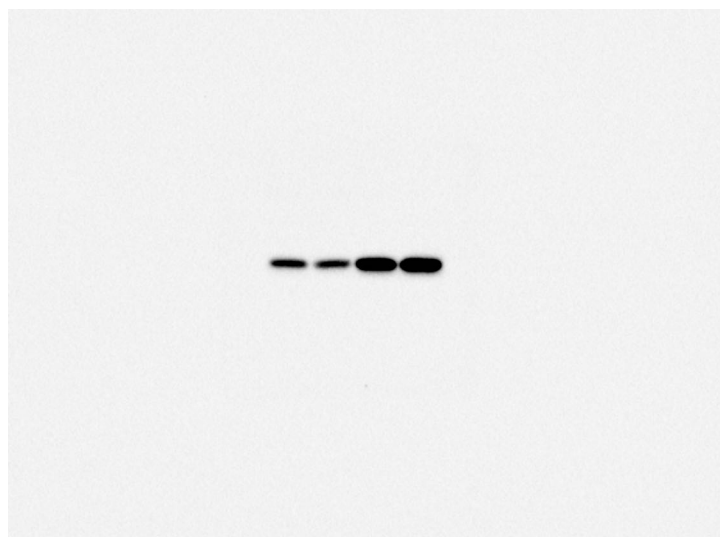

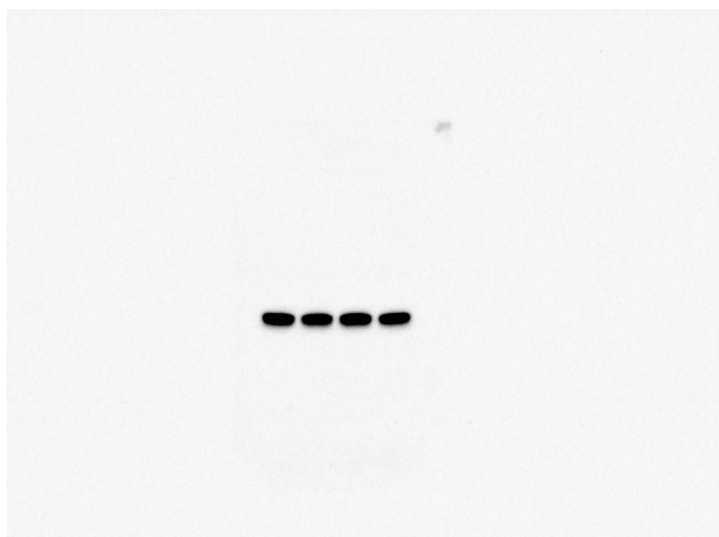

Supplement: SourceData FS4 — is the source file for Fig. S4. [file JCB_202112018_SourceDataFS4.pdf]
